# The Effect of Different Global Navigation Satellite System Methods on Positioning Accuracy in Elite Alpine Skiing

**DOI:** 10.3390/s141018433

**Published:** 2014-10-03

**Authors:** Matthias Gilgien, Jörg Spörri, Philippe Limpach, Alain Geiger, Erich Müller

**Affiliations:** 1 Department of Physical Performance, Norwegian School of Sport Sciences, Sognsveien 220, Oslo 0806, Norway; 2 Department of Sport Science and Kinesiology, University of Salzburg, Schlossallee 49, Hallein/Rif 5400, Austria; E-Mails: joerg.spoerri@sbg.ac.at (J.S.); erich.mueller@sbg.ac.at (E.M.); 3 Institute of Geodesy and Photogrammetry, ETH Zurich, Schafmattstrasse 34, HPV G 58.2, Zurich 8093, Switzerland; E-Mails: philippe.limpach@geod.baug.ethz.ch (P.L.); alain.geiger@geod.baug.ethz.ch (A.G.)

**Keywords:** positioning, kinematics, locomotion, GPS/GNSS, wearable system, navigation, photogrammetry, validation, accuracy, sport, snowsport, sports medicine

## Abstract

In sport science, Global Navigation Satellite Systems (GNSS) are frequently applied to capture athletes' position, velocity and acceleration. Application of GNSS includes a large range of different GNSS technologies and methods. To date no study has comprehensively compared the different GNSS methods applied. Therefore, the aim of the current study was to investigate the effect of differential and non-differential solutions, different satellite systems and different GNSS signal frequencies on position accuracy. Twelve alpine ski racers were equipped with high-end GNSS devices while performing runs on a giant slalom course. The skiers' GNSS antenna positions were calculated in three satellite signal obstruction conditions using five different GNSS methods. The GNSS antenna positions were compared to a video-based photogrammetric reference system over one turn and against the most valid GNSS method over the entire run. Furthermore, the time for acquisitioning differential GNSS solutions was assessed for four differential methods. The only GNSS method that consistently yielded sub-decimetre position accuracy in typical alpine skiing conditions was a differential method using American (GPS) and Russian (GLONASS) satellite systems and the satellite signal frequencies L1 and L2. Under conditions of minimal satellite signal obstruction, valid results were also achieved when either the satellite system GLONASS or the frequency L2 was dropped from the best configuration. All other methods failed to fulfill the accuracy requirements needed to detect relevant differences in the kinematics of alpine skiers, even in conditions favorable for GNSS measurements. The methods with good positioning accuracy had also the shortest times to compute differential solutions. This paper highlights the importance to choose appropriate methods to meet the accuracy requirements for sport applications.

## 1. Introduction

Global Navigation Satellite Systems (GNSS) are frequently applied to capture athletes' position, velocity and acceleration (PVA) in team sports [1,2] and individual sports, such as running/locomotion [3–15], orienteering [16], sailing/rowing [17], ski jumping [18], cross country skiing [19–21], snowboarding [22,23], and alpine skiing [24–31]

In the sport of competitive alpine skiing, there are three main demands on a valid and practicable measurement system for capturing skiers' kinematics: (1) it has to fulfill high accuracy standards in order to detect the small but substantial differences between athletes' trajectories [32,33]; (2) it should cause minimal interference with the athletes' competitive skiing [34]; (3) it should allow the largest possible capture volumes in order to be able to analyze entire competitions and/or training runs.

In order to meet these requirements, to date a number of different methods have been suggested: non-differential GNSS methods [31,35], real-time differential GNSS methods [25,36,37] and post-processed, differential GNSS methods [30,38]. While some applications use only the American GPS system [34,38], other applications combine GPS with the Russian (GLONASS) system [25,28,30]. Furthermore, some differential methods use the L1 frequency only [28], while others use frequencies L1 and L2 [25,30].

Despite the large number of GNSS applications in alpine skiing and sports in general, no study has ever comprehensively compared the available GNSS methods with respect to position accuracy. Hence, the magnitude of position accuracy of the applied methods is largely unknown. Furthermore, signal obstruction can lead to partial or total loss of satellite signals and loss of differential solutions. Due to the relatively high dynamics in alpine skiing, the time needed to acquire a differential positioning solution is crucial.

Therefore, this study assessed position accuracy and the time taken to compute differential GNSS solutions for five typical GNSS methods commonly used in sport science, in the case of alpine ski racing under three different satellite signal obstruction conditions. The goal of this study is to investigate which GNSS methods meet the accuracy requirements of dynamic sports applications in difficult satellite signal obstruction conditions, such as alpine skiing. Consequently, the study consisted of two main steps: In the first step, the position solutions were compared to an independent video-based photogrammetric reference system during one turn of a giant slalom course. In the second step, the most accurate and consistent GNSS method from Step 1 was defined as the reference method and was compared to the other methods over the larger capture volume of an entire giant slalom run. In addition, the time required to compute differential GNSS solutions was assessed for each method and condition.

## 2. Methods

### 2.1. Data Acquisition

Six male European Cup or former World Cup (WC) skiers performed two runs on a typical giant slalom course. The course consisted of twelve gates and lasted for approximately 20 s. The course was set on a north-facing slope with a flat section (approximately 12°) up to gate 5 and steep terrain for gates 6 to 12 (approximately 25°). Athletes skied in average at 17 m/s and reached accelerations of up to 4 m/s^2^. Minimal turn radius was 16 m. The athletes' head trajectory was captured during the entire run using GNSS. During turn 7 of the course the GNSS antenna was simultaneously captured by a video-based photogrammetric reference system (Figure 2). The data was collected on four different days (3 runs each day) from 29 March–1 April 2011 at approximately 0700 h (UTC time) each day. The location of the data collection was Kühtai, Austria (WGS 84 coordinates: X: 4′261′800; Y: 830′500; Z: 4′659′400). The study was approved by the Ethics Committee of the Department of Sport Science and Kinesiology at the University of Salzburg and the athletes were informed of the investigation's purpose and procedures.

### 2.2. GNSS Methods Simulation

**Figure 1.**
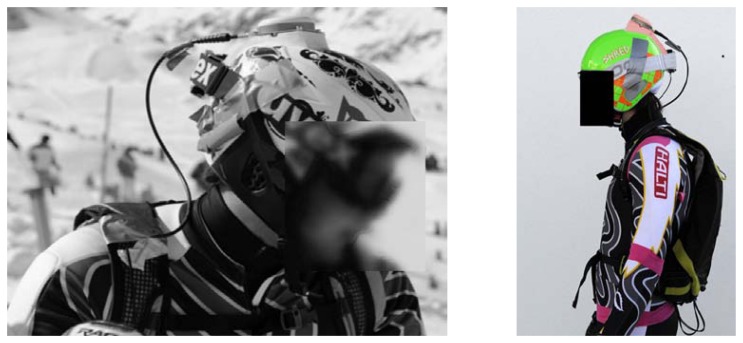
(**Left**) Magnification of the antenna installation on the skier's helmet. (Photos: Philippe Chevalier). (**Right**) An alpine skier equipped with the GNSS system. The antenna was mounted on the helmet and connected to a receiver, which was carried in a cushioned backpack.

**Figure 2.**
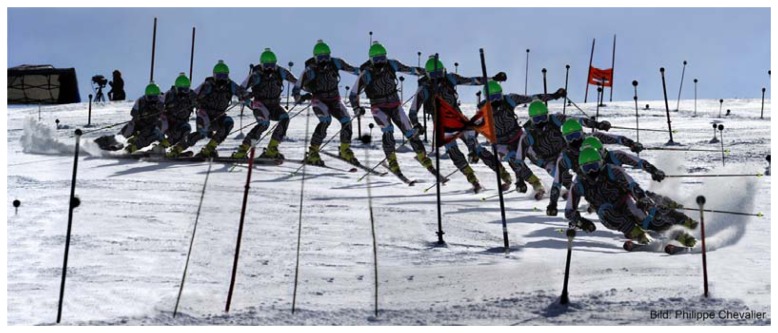
Animation of an alpine skier in the giant slalom course surrounded by the reference points of the reference measurement system at gate number 7 (Photo: Philippe Chevalier).

A GNSS antenna (Antcom G5Ant-2AT1, 160 g) was mounted on the helmet of the athlete and was connected to a GPS/GLONASS dual frequency (L1/L2) receiver (Javad Alpha-G3T, 430 g, L: 148 mm × H: 85 mm × D: 35 mm) carried in a cushioned backpack (Figure 1) and recording position signals at 50 Hz. Two GNSS base stations were mounted close to the start of the course and were equipped with antennas (Javad GrAnt-G3T) and Javad Alpha-G3T receivers.

Accurate absolute global positions of the GNSS base stations were calculated using reference data from the Austrian Positioning Service (APOS, Wien, Austria) and the geodetic post-processing software Justin (Javad). All GNSS measurements were determined in the WGS84 (Universal Transverse Mercator zone 32, Northern Hemisphere) coordinate system. The skier's GNSS antenna trajectory was computed using the geodetic post-processing software Justin in double difference mode. When the post-processing software Justin was unable to fix ambiguities (integer ambiguities), float ambiguities (real number ambiguities) were calculated using the chosen frequencies (L1 or L1 and L2) and satellite systems (GPS or GPS and GLONASS). No cascading from L1 and L2 to L1 was used.

The kinematic position solutions were computed for five different GNSS methods (Table 1). Method *A* was a differential phase solution that included both GPS and GLONASS satellite signals and the signal frequencies L1 and L2. Method *B* was similar to method *A*, but using frequency L1 only. Method *C* was similar to method *A* except that GPS signals only were used. Method *D* was similar to *C* but using frequency L1 only. Method *E* was a non-differential code solution using only GPS code signals (the software was choosing among the code signals P1, P2, C2, C5 and C/A. In order to simulate measurement environments with different grades of satellite signal obstruction, each GNSS method was computed for circular elevation angles of 10°, 30° and 40° on top of the masking caused by the topography. The elevation angles were adjusted in the post-processing software. All signals (direct or indirect) of satellites with an elevation angle smaller than the 10°, 30° or 40° cut-off elevation angle were left out from the solution computation. Hence, 5 methods were used with 3 different satellite signal obstruction conditions each. The method names are written in italic (*A*–*E*), while the position vectors of the skier trajectory are written in italic and bold (***A***–***E***).

### 2.3. Step 1

#### 2.3.1. Reference Measurement System

Turn 7 was equipped with a video-based reference system, which was used to determine the GNSS antenna trajectory independently from the GNSS methods. The reference system consisted of six panned, tilted and zoomed HDV cameras (Sony, PMW-EX3), which captured the skier at 50 Hz. The camera images were time-synchronized using an electronic gen-lock signal. The GNSS antenna was manually digitized in the camera images. Reference points were mounted along the course for turns 6, 7 and 8 and their positions were determined using a geodetic tachymeter in a local coordinate system (LCS). The reference points and the camera positions were used along with a DLT-based panning algorithm by Drenk (1994) to reconstruct the GNSS antenna trajectory (***P****_REF_*) during turn 7. The start and end of turn 7 was determined using the definition of [39]. ***P****_REF_* was low-pass filtered using a second-order Butterworth filter with a cut-off frequency determined according to the Jackson Knee method [40]. The mean resultant photogrammetric error is known to be 23 mm with a standard deviation of 10 mm [41].

#### 2.3.2. Method Comparison

**Figure 3.**
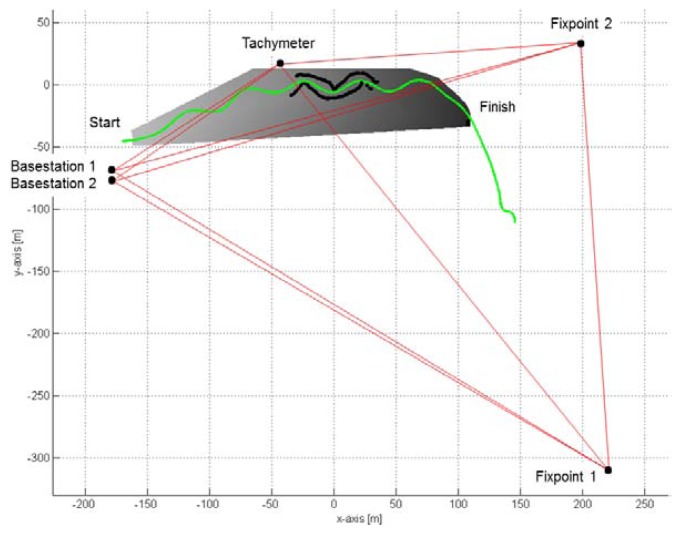
Overview of the geodetic measurement setup. Skier trajectory is drawn in green, the digital terrain model in gray. The gray scale is darker with decreasing altitude. Fixpoints 1 and 2, the tachymeter position and the GNSS base stations are marked with black dots. The geodetic network is drawn in red. The black dots along the skier trajectory represent the calibration points used for the video-based photogrammetry at turn 7. The graph is shown in the LCS.

In order to compare the GNSS-based trajectories with the reference trajectory, the GNSS coordinates in the global (WGS84) coordinate system were transformed into the local coordinate system (LCS). Five points (tachymeter position, two GNSS base stations and two reference points) surrounding the area (see Figure 3) were periodically measured with the tachymeter (LCS) and differential GNSS and were used for matching. The transformation was accomplished using a least square resection method (Sheynin, 1995) and the software Leica Geo Office (Leica Geosystems, Heerbrugg, Switzerland). The matching of the coordinate system was conducted before and after the motion capture period on all four days. The drift over time between the two measurements was accounted for by distribution of the differences between the solution before and the solution after the motion capture period by time interpolation. The mean difference of the resection at a reference point was below 13 mm in the vertical component and below 9 mm in the horizontal component, with standard deviations of 4 mm and 3 mm, respectively. The z-axis of the LCS pointed in the opposite direction to gravity, the x-axis was parallel to the vector from gate 7 to gate 9 and the y-axis was orthogonal to the x and y axis.

The GNSS antenna trajectory solutions ***A***–***E*** were compared to ***P****_REF_* every 0.02 s within turn 7 using GPS time as reference time. ***P****_REF_* was time interpolated to the GPS time points to allow spatial comparison at the corresponding point in time. The spatial differences between ***P****_REF_* and the GNSS position solutions were expressed as vector norms (XYZ) and decomposed in the z-direction (Z) and the horizontal component (XY). For the double difference methods (integer fixed and real number float ambiguity), the differences were computed for all fixed solutions and all float solutions separately but also for the combination of fixed and float solutions. For the latter, fixed solutions were used when these could be computed, while float solutions were used when the system was unable to fix ineger ambiguities. The differences of the combination of fixed and float solutions was computed as a measure for the overall performance of the system, while the comparison of only fixed or only float solutions were computed to give more detailed insight in the system's performance. For each run the mean difference, standard deviation (SD) of the difference, the median of the difference and the maximal difference (maximum) were calculated. Subsequent to the analysis of each trajectory, these measures were averaged across the 12 runs for each component. To express the results for the methods ***A***–***E*** in a histogram, the difference of each epoch to the reference system was assigned to accuracy categories and expressed as fractions of the total time the skier skied through the reference turn. The timespans in which the methods were unable to compute solutions were marked as NaN, along with the spatial differences for fixed and float solutions. In addition the number of runs for which a fixed solution was computed at least once was computed. In addition, the timespan when fixed, float or no solutions that were computed were expressed as % of total time the skier skied through the reference turn.

### 2.4. Step 2

#### 2.4.1. Position Accuracy Assessment

In Step 2, the same GNSS data as in Step 1 were used, but, in contrast, the data from the entire run (12 turns) were assessed. The analysis of the spatial differences started shortly after the start gate when the individual skiers reached a speed of 2 m/s (using the Doppler-speed measurement of method *A*). The analysis ended when the skiers passed the last gate. The analysis in Step 1 revealed that method *A* under the condition 10° (***A_10_***) was the most accurate and consistent (see results Step 1). Consequently, ***A_10_*** served as the reference method in Step 2 using the same approach as in Step 1, the spatial differences between ***A***–***E*** and ***A_10_*** were calculated each 0.02 s from start to finish for the elevation masks 10°, 30° and 40°. GNSS measurements of the unit on the athletes were started (warm start) on average 69 s (minimum 53 s, maximum 92 s) before skiers reached the speed of 2 m/s, while the measurement on the base station unit was started earlier. Between the start of the GNSS measurement and the time point when skiers left the start gate, skiers stood in an upright position in an open area with no obstacles other than the topography affecting satellite signal reception. The same statistics as in Step 1 were used to characterize the spatial differences between ***A***–***E*** and ***A_10_***.

#### 2.4.2. Assessment of Time to Fix Ambiguities for the Positioning Solution

The time to fix ambiguities (integer ambiguities) for the positioning solution was assessed at measurement start. The time, from the start of the GNSS measurement to the instant when integer ambiguities were fixed, was calculated for methods *A* to *D* (method *E* was a non-differential code solution). The mean and SD of the times to fix integer ambiguities was calculated for the 12 trials.

## 3. Results and Discussion

### 3.1. Results

#### 3.1.1. Step 1

The results in Tables 2 and 3 show that: (a) method *A* was the only method with fixed integer ambiguities in all elevation angle conditions; (b) All differential methods (*A*–*D*) had fixed ambiguities in the 10° condition; and (c) the number of trajectories with fixed ambiguities was substantially reduced for methods *D* in the 30° and methods *B*–*D* in the 40° condition. Table 3 shows that method *A* had float ambiguities (real number ambiguities) for 8% of the time but as the only lacked time periods without solution (NaN). In the 40° condition methods *B*–*E* had substantial periods of time without solutions (NaN). Comparing method *B* and *C* in the 40° condition, method *B* managed to compute float (real number ambiguities) for a larger period of time, reducing the periods with no solution substantially compared to method *C*.

Table 4 shows the he system's overall performance, the combination of fixed and float solutions for the differential methods *A*–*D*, hence the position differences between ***A***–***E*** and ***P****_REF_* (mean, median, maximum and SD). Examining the mean and median of the position differences in Table 4 reveals that: (a) the position differences generally increased with increasing elevation angle; (b) the position differences (mean and median) and SD were larger in the Z dimension than in the XY dimension; (c) method *E* had the largest position difference mean, median, SD and maximal difference of all methods in all conditions; (d) in the conditions 10° and 30° the position differences (mean and median) were smallest for method *A*, followed by *C*, *B*, *D* and *E*; (e) the differences (mean and median) in the condition 40° were smallest for method *A*, followed by *C*, *B* and *E*, while method *D* was unable to compute a differential solution; (f) method *A* was the only method with mean and median XYZ differences smaller than 7 cm in all elevation angle conditions; (g) in the 10° condition methods *A*–*C* had mean and median XYZ differences smaller than 2 cm and SDs smaller than 4 cm.

**Figure 4.**
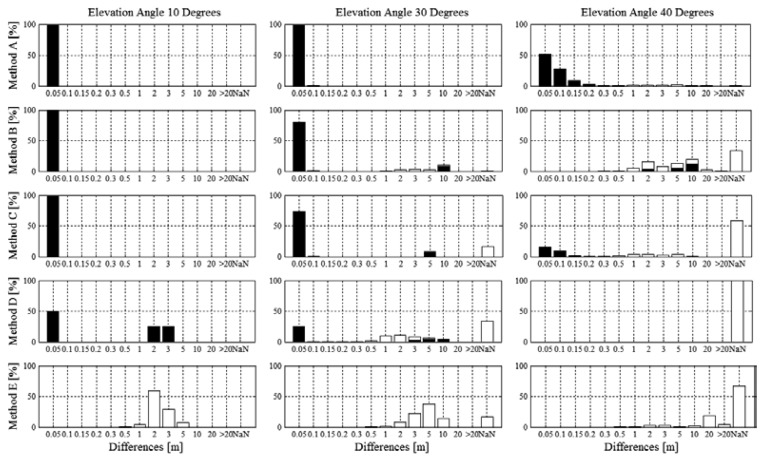
Histograms of the position difference norms (XYZ) between the trajectories of methods *A*–*E* (*A_1_*–*E_1_*) and the reference trajectory (*P_REF_*) for the elevation masking conditions 10°, 30° and 40°. On the horizontal axis, NaN indicates the amount of time when no solution could be computed. Black bars indicate fixed solutions, white bars indicate float solutions. NaN indicates that the system was unable to compute a solution.

The histograms shown in Figure 4 illustrate the norm of the spatial differences (XYZ) for fixed integer ambiguity (black bars) and real number float ambiguity (white bars) solutions along with the proportions of time when no solutions could be computed with the respective methods (NaN, white bars). For the condition of 10°, all differential methods were able to fix integer ambiguities for the entire reference turn. Method *D* consisted of differences smaller than 5 cm and in the range of 2–3 m to 50% of total time each. Integer ambiguity was fixed for the entire time and hence, the differences in the order of meters must have occurred due to inappropriate integer fixing. Method *E* had no differences smaller than 0.5 m or larger than 10m. For the condition of 30°, method *A* had only fixed integer ambiguity solutions and position difference mean and median were smaller than 5 cm. The fraction of the data without solution (NaN) was largest for method *D*, followed by *E*, *C* and *B*. For method *B* and *D* approximately 80% and 70% of the differences respectively were smaller than 5cm, while the remainders were in the order of meters. The reasons for the differences in the order of meters were caused both by sections of real number float ambiguity and sections of inappropriate integer fixing. Method *E* had most differences in the range of 2–15 m. For the condition of 40°, the fraction of the data without solution (NaN) was largest for method *D* (no solution) followed by *E*, *C*, *B* and *A*. Method *A* had differences smaller than 10 cm for more than 75% of the time. Method *B* had most differences in the range of 1–10 m. Compared to method *B*, method *C* had a larger fraction with differences smaller than 0.5 m and periods of no solution. Method B had a larger fraction of inappropriate integer fixing. Method *E* had no solution for about 70% of the time and all differences were larger than 1 m. Table 5 shows the statistics of the differences for the periods when integer fixed ambiguity solutions were computed and Table 6 shows the statistics of the differences for the periods when real number float ambiguity solutions were computed.

#### 3.1.2. Step 2

The timespans from (warm) start of the differential GNSS measurement until ambiguities were fixed are given in the right hand side of Table 7. The mean time to fix integer ambiguities was below 1.2 s for method *A* in all conditions and for method *B* and *C* in the 10° condition. Method *C* required less time (mean) to fix the integer ambiguities than method *B*. Method *D* was slowest to fix integer ambiguities and was unable to do so in the 40° condition.

**Figure 5.**
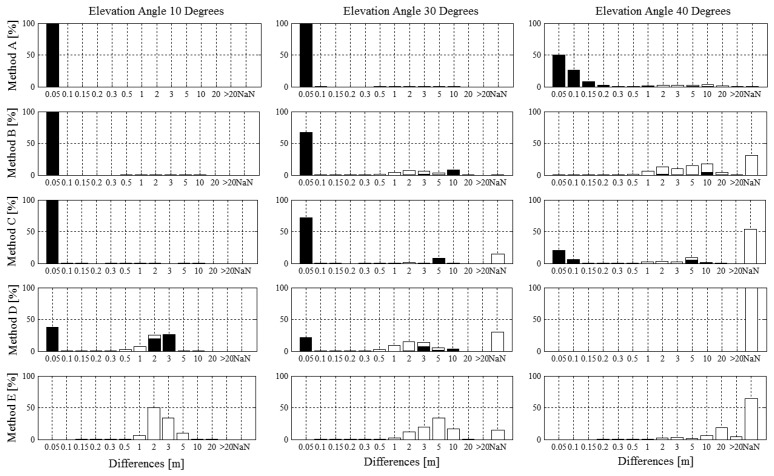
Results of study 2: Histograms of the position difference norms (XYZ) between the trajectories of methods *A*–*E* (*A*–*E*) and the reference trajectory (*A_10_*) for the elevation masking conditions 10°, 30° and 40°. NaN on the horizontal axis indicates the amount of time when no solution could be computed. Black bars indicate fixed solutions, white bars indicate float solutions. NaN indicates that the system was unable to compute a solution.

Tables 8, 9, 10 and 11 and Figure 5 indicate that the spatial differences of the combination of integer fixed ambiguity and real number float ambiguity solutions for method *A*–*D* (Table 9, Figure 5), the code solution of (method *E*) and the separate differences for integer fixed ambiguity (Table 10) and real number float ambiguity solutions (Table 11) in Step 2 were comparable to the results of study 1 (Tables 4, 5 and 6), where the analysis was based on a specific section only. Considering the entire run, a larger number of trajectories managed to compute a fixed integer ambiguities solution at least once during the run (Table 7, left side) but the fraction of time during which integer fixed ambiguity could be computed were in general slightly reduced.

### 3.2. Discussion

The current study produced the following main findings: (1) For the periods when solutions were computed the spatial differences were smallest for method *A*, followed by *C*, *B*, *D* and *E*; (2) The period of time for which no differential solution could be computed was shortest for method *A* and increased for methods *B*, *C* and *D* respectively; (3) Methods *A*, *B* and *C* were approximately equally accurate for the 10° condition; (4) Time to fix integer ambiguities was shortest for method *A* followed by methods *C*, *B* and *D*. The elevation angles 10°, 30° and 40° were chosen to simulate realistic signal obstruction conditions for WC alpine ski racing. Positioning dilution of precision (PDOP) was determined by forerunners skiing 19 real WC races, using method *A*. The measurements in the WC races revealed that PDOP of 1.5 to 2.0 occurred 76% of the time, PDOP between 2 and 5 for 19% of the time and PDOP above 5 for approximately 5% of the time for method *A*. These measures are in agreement with the findings of other studies [28,42]. During the experiment for this simulation a mean PDOP of 1.9 was found for an elevation angle of 30° and a mean PDOP of 7.8 for the 40° elevation angle condition. Hence, the 10° elevation angle condition represented conditions without any additional obstruction, as would be the case on glaciers for example, while the 30° and 40° conditions represented ordinary and extreme WC racing conditions, respectively.

In order to be able to detect relevant functional differences, the accuracy requirements for position data in alpine skiing must be in the range of a few centimeters in slalom and giant slalom [33], but might be larger in the speed disciplines super-G and downhill. Hence, the results of study 1 and 2 showed that GNSS measurements in WC races should be accomplished using both GPS and GLONASS and frequency L1 and L2 (method *A*). If measuring in areas with little satellite signal obstruction, for example on glaciers, GLONASS or frequency L2 can probably be dropped, if high-end devices are used. However, under real WC competition conditions, where topography and trees are obstructing the satellite signal, both accuracy of the differential (fixed and float) solutions and the amount of differential solutions decreased, if either frequency L2 (method *B*) or GLONASS (method *C*) was dropped. Comparing method *B* and *C*, the use of frequency L2 increased the accuracy of the integer fixed solutions in method *C* compared to method *D*, possibly as a result of the reduction of disturbances in the ionosphere [42]. However, using method C the share of data with no solution was increased compared to method *B*. The increased amount of differential solutions for method *B* compared to method *C* might be the result of a better satellite signal constellation since geometrical dilution of precision (GDOP) was increased for method *B*. The effects of satellite signal frequencies and satellite systems on the share of fixed integer ambiguities and accuracy found in this study are in line with the literature [43].

Figures 4 and 5 show that a certain amount of measurements with methods *B*–*D* resulted in differences in the order of meters consisting both of integer fixed ambiguity and real number float ambiguity solutions. For the cases when large differences occurred with fixed integer ambiguity solutions might be a result of inappropriate integer ambiguity fixing and probably be a result of cycle slips. Time series analysis showed that in most of these cases the integer fixed solutions were wrong for the entire trajectory. Taking method *D* in condition 10° as an example the ambiguities of 6 trajectories were properly fixed causing small differences but the remaining six trajectories were inappropriately fixed, causing differences in the order of meters. Hence, in such cases, ambiguities were often wrongly fixed while the skier was still static prior to the start and ambiguity status did not change from fixed to float or vice versa during skiing. A reason for the inappropriate integer ambiguity fixing might be the Kalman filter adjustment to a dynamic mode that was undertaken for this study to allow tracking of the dynamic application. The dynamic mode might reduce the number of constraints and lead to difficulties in identifying cycle slips. However, the study also showed, that adding the satellite system GLONASS and frequency L2 enabled the software to identify cycle slips more easily.

**Figure 6.**
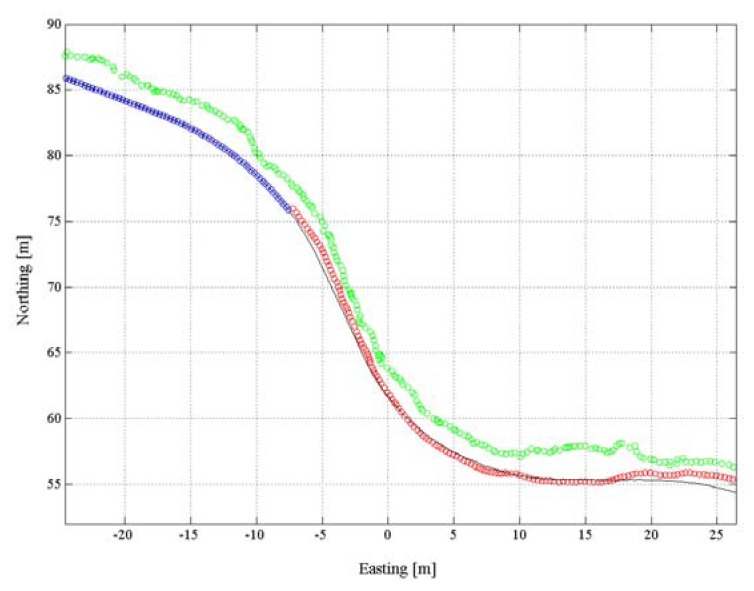
A trajectory plotted for 2 turns from the birds-eye perspective. In green the standalone code position solution *E*, in blue the positions for the period when the software managed to fix integer ambiguities, in red the positions for the period where only real number float ambiguities could be computed. The position solution for the reference method ***A****_10_* is plotted as a black line illustrating the characteristics of the spatial differences between the different types of GNSS solutions.

Figure 6 shows an example of a trajectory for which the ambiguity mode shifted from real number float ambiguity to integer fixed ambiguity mode during a run. The red circles show the positions of the first part of the turn, with real number float ambiguities and the blue circles show the section with integer fixed ambiguities. The black line represents the reference trajectory and the standalone code solution *E* is plotted in green. The example in Figure 6 illustrates the typical size and nature of the differences to the reference trajectory for the real number float ambiguity and the standalone code solution. The standalone code solution *E* shows increased systematic and random error compared to the differential real number float solution.

Since GNSS signal obstruction can lead to loss of differential solutions, the time to fix integer ambiguities and quickly regain acceptable accuracy is crucial [38]. This study showed that time to fix integer ambiguities was shorter when both frequencies (L1 and L2) were used instead of L1 only. Hence, rapid integer ambiguity fixing is a second reason to use frequency L1 and L2 when measuring alpine ski racing under ordinary conditions.

However, it is known that time to fix integer ambiguities also depends on the GNSS equipment [38] and might deviate when different GNSS devices are used. If different receivers, antenna and processing software are applied, positioning accuracy may also differ, especially if low-cost devices are used. Initial comparison of standalone low-cost devices applying L1 and GPS resulted in substantially larger differences to method *A* than the ones presented for method *E* in this study.

The findings of this study might not only be applicable to the sport of alpine skiing, but also to other sports held in surroundings with variable GNSS signal obstruction. Furthermore, the results of the 10° condition might be applicable to all kinds of sports allowing favorable GNSS measurement conditions. Buildings, vegetation and topography might mask satellite signals in a manner, which is comparable to the conditions known in alpine ski racing (30° and 40° condition). Hence, a broad variety of GNSS operators might benefit from an enhanced understanding of the significance of GNSS methods on their outcomes.

### 3.3. Limitations

The current study has several limitations, which may influence the results of the method comparisons: (1) the accuracy of the photogrammetric reference system was found to be 23 mm ± 10 mm for well-defined points. Additional uncertainty might be added in the digitalization of the antenna GNSS antenna center; (2) The resection method used for the matching of the reference system with the GNSS measurements had a mean difference of 16 mm. The SD of the difference was ±5 mm; (3) The reference system of Step 2 is not independent. It is therefore theoretically possible that method A had a larger difference from the true trajectory than one of the other methods. However, the reference method was chosen as it allowed assessing how the GNSS system performed for the true dynamics of alpine ski racing with its high accelerations and antenna tilt kinematics in the typical surrounding of alpine skiing. The used reference method however was accurate enough to measure and highlight the differences between methods as most of them are either on the level of cm or m. To assess smaller differences between methods, other reference systems should be used, which likely do not allow the assessment of unconstrained competitive alpine skiing.

## 4. Conclusions

The only GNSS method consistently yielding sub-decimeter position accuracy in typical alpine skiing conditions was a differential method using GPS and GLONASS satellite systems and the satellite signal frequencies L1 and L2. In conditions with minimal satellite signal obstruction, valid results were also found if either the satellite system GLONASS or frequency L2 was dropped from the best configuration. All other methods failed to meet the accuracy requirements for alpine skiing even in conditions favorable for GNSS measurements. The methods with good positioning accuracy had also the shortest times to compute differential solutions.

## Figures and Tables

**Table 1.** Composition of the 5 GNSS methods applied in study 1 and 2.

**Method**	**GNSS**		**Frequency**		**Processing**	
		
	**GPS**	**GLONASS**	**L1**	**L2**	**Differential**	**Non-Differential**
*A*	X	X	X	X	X	
*B*	X	X	X		X	
*C*	X		X	X	X	
*D*	X		X		X	
*E*	X		Code			X

**Table 2.** Number of trajectories with fixed positioning solutions when passing through the reference area. The total number of trajectories was 12.

**Elev. Angle**	**# of Trajectories with Fixed Solutions**		

	**10°**	**30°**	**40°**
Method *A*	12	12	12
Method *B*	12	11	2
Method *C*	12	10	3
Method *D*	12	5	-
Method *E*	-	-	-

**Table 3.** The time during which fixed or float ambiguities (method *A*–*D*), code solution (method *E*) or no solution (NaN) was computed are given in % of the time the skiers used to ski through the reference turn.

**Elev. Angle**	**Fixed**			**Float (Method *A*–*D*)** **Code (Method *E*)**			**No Solution (NaN)**		
		
	**10°**	**30°**	**40°**	**10°**	**30°**	**40°**	**10°**	**30°**	**40°**
Method *A*	100	100	92	0	0	8	0	0	0
Method *B*	100	90	20	0	10	46	0	0	34
Method *C*	100	83	25	0	0	16	0	17	59
Method *D*	100	36	0	0	31	0	0	34	100
Method *E*				100	83	33	0	17	67

**Table 4.** Mean, Median, SD and Maximum of the spatial differences between the skier trajectories *fixed and float* solutions (*A_1_*–*D_1_*), the code solution *E_1_* and the reference trajectory (*P_REF_*) for the turn with the independent reference system. XYZ represents the difference norm in 3 dimensions, XY the difference norm in the horizontal plane and Z in the vertical direction. NaN indicates that the method was unable to compute solutions for the given condition.

**Elev. Angle**	**Mean XYZ [m]**			**Mean XY [m]**			**Mean Z [m]**		
		
	**10°**	**30°**	**40°**	**10°**	**30°**	**40°**	**10°**	**30°**	**40°**

Method *A*	0.00	0.02	0.30	0.00	0.01	0.09	0.00	0.01	0.28
Method *B*	0.01	1.02	4.28	0.00	0.48	1.27	0.00	0.90	3.99
Method *C*	0.01	0.47	0.82	0.00	0.34	0.27	0.00	0.33	0.76
Method *D*	0.95	1.35	NaN	0.48	0.44	NaN	0.78	1.18	NaN
Method *E*	1.88	3.6	12.22	1.09	1.92	3.17	1.32	2.74	11.72

**Elev. Angle**	**Median XYZ [m]**			**Median XY [m]**			**Median Z [m]**		
		
	**10°**	**30°**	**40°**	**10°**	**30°**	**40°**	**10°**	**30°**	**40°**

Method *A*	0.00	0.01	0.05	0.00	0.01	0.01	0.00	0.01	0.05
Method *B*	0.01	0.02	3.44	0.00	0.01	1.21	0.00	0.02	3.32
Method *C*	0.01	0.01	0.07	0.00	0.01	0.02	0.00	0.01	0.07
Method *D*	0.02	0.75	NaN	0.02	0.33	NaN	0.02	0.39	NaN
Method *E*	1.73	3.43	13.54	1.11	1.69	3.00	1.13	2.50	13.03

**Elev. Angle**	**SD XYZ [m]**			**SD XY [m]**			**SD Z [m]**		
		
	**10°**	**30°**	**40°**	**10°**	**30°**	**40°**	**10°**	**30°**	**40°**

Method *A*	0.00	0.01	1.27	0.00	0.00	0.36	0.00	0.01	1.22
Method *B*	0.00	2.40	3.49	0.00	1.19	0.76	0.00	2.09	3.52
Method *C*	0.00	1.35	1.38	0.00	0.98	0.44	0.00	0.93	1.31
Method *D*	1.00	1.63	NaN	0.55	0.47	NaN	0.87	1.63	NaN
Method *E*	0.66	1.43	6.80	0.39	1.05	1.83	0.94	1.64	6.70

**Elev. Angle**	**Maximal XYZ [m]**			**Maximal XY [m]**			**Maximal Z [m]**		
		
	**10°**	**30°**	**40°**	**10°**	**30°**	**40°**	**10°**	**30°**	**40°**

Method *A*	0.00	0.06	4.28	0.00	0.02	1.01	0.00	0.05	4.16
Method *B*	0.02	8.07	26.90	0.01	4.21	5.04	0.02	7.50	26.48
Method *C*	0.03	4.50	5.84	0.02	3.25	1.68	0.03	3.11	5.60
Method *D*	2.44	5.04	NaN	1.41	1.80	NaN	2.17	5.03	NaN
Method *E*	4.53	9.27	24.67	3.02	5.33	7.85	4.20	9.15	24.40

**Table 5.** Mean, Median, SD and Maximum of the spatial differences between the skier trajectories *fixed* solutions (*A_1_*–*E_1_*) sand the reference trajectory (*P_REF_*) for the turn with the independent reference system. XYZ represents the difference norm in 3 dimensions, XY the difference norm in the horizontal plane and Z in the vertical direction—indicates that the method was unable to compute fixed solutions for the given condition.

**Elev. Angle**	**Mean XYZ [m]**			**Mean XY [m]**			**Mean Z [m]**		
		
	**10°**	**30°**	**40°**	**10°**	**30°**	**40°**	**10°**	**30°**	**40°**

Method *A*	0.00	0.02	0.05	0.00	0.01	0.01	0.00	0.01	0.05
Method *B*	0.01	0.69	1.63	0.00	0.36	0.58	0.00	0.59	1.48
Method *C*	0.01	0.47	0.03	0.00	0.34	0.01	0.00	0.33	0.03
Method *D*	0.95	0.70	-	0.48	0.11	-	0.78	0.67	-

**Elev. Angle**	**Median XYZ [m]**			**Median XY [m]**			**Median Z [m]**		
		
	**10°**	**30°**	**40°**	**10°**	**30°**	**40°**	**10°**	**30°**	**40°**

Method *A*	0.00	0.01	0.04	0.00	0.01	0.01	0.00	0.01	0.04
Method *B*	0.01	0.02	0.00	0.00	0.01	0.00	0.00	0.02	0.00
Method *C*	0.01	0.01	0.02	0.00	0.01	0.01	0.00	0.01	0.01
Method *D*	0.02	0.01	-	0.02	0.00	-	0.02	0.00	-

**Elev. Angle**	**SD XYZ [m]**			**SD XY [m]**			**SD Z [m]**		
		
	**10°**	**30°**	**40°**	**10°**	**30°**	**40°**	**10°**	**30°**	**40°**

Method *A*	0.00	0.01	0.04	0.00	0.00	0.01	0.00	0.01	0.04
Method *B*	0.00	2.22	2.77	0.00	1.16	0.94	0.00	1.89	2.63
Method *C*	0.00	1.35	0.03	0.00	0.98	0.01	0.00	0.93	0.03
Method *D*	1.00	1.67	-	0.55	0.33	-	0.87	1.65	-

**Elev. Angle**	**Maximal XYZ [m]**			**Maximal XY [m]**			**Maximal Z [m]**		
		
	**10°**	**30°**	**40°**	**10°**	**30°**	**40°**	**10°**	**30°**	**40°**

Method *A*	0.00	0.06	0.27	0.00	0.02	0.05	0.00	0.05	0.26
Method *B*	0.02	8.07	9.41	0.01	4.21	3.18	0.02	6.88	8.86
Method *C*	0.03	4.50	0.16	0.02	3.25	0.05	0.03	3.11	0.16
Method *D*	2.44	5.04	-	1.41	1.80	-	2.17	5.03	-

**Table 6.** Mean, Median, SD and Maximum of the spatial differences between the skier trajectories *float* solutions (*A_1_*–*E_1_*) and the reference trajectory (*P_REF_*) for the turn with the independent reference system. XYZ represents the difference norm in 3 dimensions, XY the difference norm in the horizontal plane and Z in the vertical direction—indicates that the method did not compute float solutions for the given condition.

**Elev. Angle**	**Mean XYZ [m]**			**Mean XY [m]**			**Mean Z [m]**		
		
	**10°**	**30°**	**40°**	**10°**	**30°**	**40°**	**10°**	**30°**	**40°**

Method *A*	-	-	0.25	-	-	0.08	-	-	0.23
Method *B*	-	0.33	2.64	-	0.12	0.69	-	0.30	2.50
Method *C*	-	-	0.78	-	-	0.26	-	-	0.73
Method *D*	-	0.64	-	-	0.33	-	-	0.51	-

**Elev. Angle**	**Median XYZ [m]**			**Median XY [m]**			**Median Z [m]**		
		
	**10°**	**30°**	**40°**	**10°**	**30°**	**40°**	**10°**	**30°**	**40°**

Method *A*	-	-	0.00	-	-	0.00	-	-	0.00
Method *B*	-	0.00	1.56	-	0.00	0.63	-	0.00	1.36
Method *C*	-	-	0.00	-	-	0.00	-	-	0.00
Method *D*	-	0.00	-	-	0.00	-	-	0.00	-

**Elev. Angle**	**SD XYZ [m]**			**SD XY [m]**			**SD Z [m]**		
		
	**10°**	**30°**	**40°**	**10°**	**30°**	**40°**	**10°**	**30°**	**40°**

Method *A*	-	-	1.28	-	-	0.36	-	-	1.23
Method *B*	-	1.14	3.62	-	0.39	0.70	-	1.07	3.59
Method *C*	-	-	1.39	-	-	0.45	-	-	1.32
Method *D*	-	0.88	-	-	0.42	-	-	0.80	-

**Elev. Angle**	**Maximal XYZ [m]**			**Maximal XY [m]**			**Maximal Z [m]**		
		
	**10°**	**30°**	**40°**	**10°**	**30°**	**40°**	**10°**	**30°**	**40°**

Method *A*	-	-	16.81	-	-	4.46	-	-	16.21
Method *B*	-	7.88	26.90	-	2.72	5.04	-	7.50	26.48
Method *C*	-	-	5.84	-	-	1.68	-	-	5.60
Method *D*	-	3.49	-	-	1.78	-	-	3.24	-

**Table 7.** The number of trajectories (out of 12) for which a fixed ambiguities solution was computed at least once during the run is shown on the left. The duration from GNSS measurement start to the instant a fixed ambiguities solution was computed for the first time is shown on the right.

**Elev. Angle**	**Number of Trajectories with Fixed Solution**			**Time to Fix [s] Mean ± SD**		
	
	**10°**	**30°**	**40°**	**10°**	**30°**	**40°**
Method *A*	12	12	12	0.06 ± 0.10	0.06 ± 0.06	0.56 ± 1.52
Method *B*	12	12	9	1.1 ± 1.1	13.58 ± 20.50	43.70 ± 20.24
Method *C*	12	10	7	0.34 ± 0.88	4.62 ± 11.12	27.94 ± 66.18
Method *D*	12	8	-	19.96 ± 2.26	51.54 ± 46.54	-
Method *E*	-	-	-	-	-	-

**Table 8.** The fraction of the entire run time during which fixed or float ambiguities (method *A*–*D*), code solution (method *E*) or no solution (NaN) was computed are given in % of the entire run time.

**Elev. Angle**	**Fixed**			**Float (Method *A*-*D*)** **Code (Method *E*)**			**No Solution (NaN)**		
		
	**10°**	**30°**	**40°**	**10°**	**30°**	**40°**	**10°**	**30°**	**40°**
Method *A*	100	100	87	0	0	13	0	0	0
Method *B*	99	78	8	1	22	61	0	0	31
Method *C*	100	81	33	0	4	14	0	15	54
Method *D*	82	33	0	18	37	0	0	30	100
Method *E*				100	85	36	0	15	64

**Table 9.** Mean, Median, SD and Maximum of the spatial differences between the skier trajectories *fixed and float* solutions for method *A_2_*–*D_2_*, the *code* solution *E_2_* and the solution of method *A* in the condition 10°. XYZ represents the difference norm in 3 dimensions, XY the difference norm in the horizontal plane and Z in the vertical direction.

**Elev. Angle**	**Mean XYZ [m]**			**Mean XY [m]**			**Mean Z [m]**		
		
	**10°**	**30°**	**40°**	**10°**	**30°**	**40°**	**10°**	**30°**	**40°**

Method *A*	-	0.02	0.67	-	0.01	0.19	-	0.02	0.63
Method *B*	0.02	1.10	4.23	0.01	0.59	1.22	0.01	0.89	3.98
Method *C*	0.01	0.51	1.29	0.01	0.35	0.64	0.01	0.36	1.06
Method *D*	1.06	1.43	NaN	0.57	0.59	NaN	0.85	1.19	NaN
Method *E*	1.99	3.58	11.75	1.09	1.77	3.04	1.45	2.84	11.27

**Elev. Angle**	**Median XYZ [m]**			**Median XY [m]**			**Median Z [m]**		
		
	**10°**	**30°**	**40°**	**10°**	**30°**	**40°**	**10°**	**30°**	**40°**

Method *A*	-	0.02	0.05	-	0.00	0.01	-	0.01	0.04
Method *B*	0.01	0.03	3.12	0.01	0.01	1.03	0.01	0.02	3.04
Method *C*	0.01	0.02	0.06	0.01	0.01	0.01	0.01	0.01	0.06
Method *D*	1.15	1.13	NaN	0.41	0.40	NaN	0.65	0.89	NaN
Method *E*	1.87	3.40	11.91	1.06	1.53	2.96	1.32	2.59	11.39

**Elev. Angle**	**SD XYZ [m]**			**SD XY [m]**			**SD Z [m]**		
		
	**10°**	**30°**	**40°**	**10°**	**30°**	**40°**	**10°**	**30°**	**40°**

Method *A*	-	0.18	2.27	-	0.11	0.61	-	0.15	2.19
Method *B*	0.17	2.23	3.80	0.12	1.21	0.90	0.12	1.89	3.78
Method *C*	0.14	1.34	1.88	0.11	0.96	0.1.06	0.09	0.94	1.59
Method *D*	0.98	1.45	NaN	0.59	0.61	NaN	0.85	1.41	NaN
Method *E*	0.78	1.69	6.83	0.52	1.11	1.71	1.02	1.78	6.74

**Elev. Angle**	**Maximum XYZ [m]**			**Maximum XY [m]**			**Maximum Z [m]**		
		
	**10°**	**30°**	**40°**	**10°**	**30°**	**40°**	**10°**	**30°**	**40°**

Method *A*	-	5.76	8.18	-	4.03	5.43	-	4.16	6.12
Method *B*	5.78	17.88	50.20	4.03	11.70	8.09	4.18	16.78	49.68
Method *C*	5.78	5.76	10.26	4.03	4.03	4.03	4.18	5.49	9.64
Method *D*	8.14	6.13	NaN	5.40	3.84	NaN	6.12	5.47	NaN
Method *E*	10.36	13.36	37.66	7.60	9.61	11.83	7.60	12.88	37.02

**Table 10.** Mean, Median, SD and Maximum of the spatial differences between the skier trajectories *fixed* solutions for method *A_2_*–*D_2_* and the solution of method *A* in the condition 10°. XYZ represents the difference norm in 3 dimensions, XY the difference norm in the horizontal plane and Z in the vertical direction.

**Elev. Angle**	**Mean XYZ [m]**			**Mean XY [m]**			**Mean Z [m]**		
		
	**10°**	**30°**	**40°**	**10°**	**30°**	**40°**	**10°**	**30°**	**40°**

Method *A*	0.00	0.02	0.05	0.00	0.01	0.01	0.00	0.01	0.05
Method *B*	0.01	0.66	0.53	0.01	0.36	0.16	0.01	0.55	0.49
Method *C*	0.01	0.44	0.54	0.01	0.32	0.39	0.01	0.30	0.37
Method *D*	0.90	0.54	-	0.48	0.20	-	0.73	0.43	-

**Elev. Angle**	**Median XYZ [m]**			**Median XY [m]**			**Median Z [m]**		
		
	**10°**	**30°**	**40°**	**10°**	**30°**	**40°**	**10°**	**30°**	**40°**

Method *A*	0.00	0.01	0.04	0.00	0.00	0.01	0.00	0.01	0.04
Method *B*	0.01	0.02	0.00	0.00	0.01	0.00	0.00	0.01	0.00
Method *C*	0.01	0.01	0.02	0.00	0.01	0.01	0.00	0.01	0.02
Method *D*	0.01	0.00	-	0.01	0.00	-	0.01	0.00	-

**Elev. Angle**	**SD XYZ [m]**			**SD XY [m]**			**SD Z [m]**		
		
	**10°**	**30°**	**40°**	**10°**	**30°**	**40°**	**10°**	**30°**	**40°**

Method *A*	0.00	0.14	0.14	0.00	0.10	0.10	0.00	0.09	0.10
Method *B*	0.14	2.08	1.91	0.10	1.11	0.57	0.09	1.76	1.83
Method *C*	0.14	1.30	1.41	0.10	0.95	1.05	0.09	0.89	0.95
Method *D*	1.04	1.33	-	0.60	0.53	-	0.88	1.24	-

**Elev. Angle**	**Maximal XYZ [m]**			**Maximal XY [m]**			**Maximal Z [m]**		
		
	**10°**	**30°**	**40°**	**10°**	**30°**	**40°**	**10°**	**30°**	**40°**

Method *A*	0.00	5.76	5.74	0.00	4.03	4.03	0.00	4.16	4.13
Method *B*	5.78	8.12	13.25	4.03	4.27	3.18	4.18	6.95	12.90
Method *C*	5.78	5.76	5.74	4.03	4.03	4.03	4.18	4.16	4.12
Method *D*	8.14	5.07	-	5.40	1.80	-	6.12	5.06	-

**Table 11.** Mean, Median, SD and Maximum of the spatial differences between the skier trajectories *float* solutions for method *A_2_*–*D_2_* and the solution of method *A* in the condition 10°. XYZ represents the difference norm in 3 dimensions, XY the difference norm in the horizontal plane and Z in the vertical direction.

**Elev. Angle**	**Mean XYZ [m]**			**Mean XY [m]**			**Mean Z [m]**		
		
	**10°**	**30°**	**40°**	**10°**	**30°**	**40°**	**10°**	**30°**	**40°**

Method *A*	-	0.01	0.63	-	0.00	0.19	-	0.01	0.59
Method *B*	0.01	0.44	3.70	0.00	0.23	1.06	0.01	0.33	3.48
Method *C*	0.00	0.08	0.75	0.00	0.04	0.25	0.00	0.07	0.70
Method *D*	0.17	0.89	-	0.10	0.39	-	0.12	0.76	-

**Elev. Angle**	**Median XYZ [m]**			**Median XY [m]**			**Median Z [m]**		
		
	**10°**	**30°**	**40°**	**10°**	**30°**	**40°**	**10°**	**30°**	**40°**

Method *A*	-	0.00	0.00	-	0.00	0.00	-	0.00	0.00
Method *B*	0.00	0.00	0.89	0.00	0.00	2.67	0.00	0.00	2.51
Method *C*	0.00	0.00	0.00	0.00	0.00	0.00	0.00	0.00	0.00
Method *D*	0.00	0.18	-	0.00	0.47	-	0.00	0.13	-

**Elev. Angle**	**SD XYZ [m]**			**SD XY [m]**			**SD Z [m]**		
		
	**10°**	**30°**	**40°**	**10°**	**30°**	**40°**	**10°**	**30°**	**40°**

Method *A*	-	0.12	2.28	-	0.03	0.60	-	0.12	2.20
Method *B*	0.09	1.10	3.84	0.06	0.63	0.91	0.07	0.92	3.79
Method *C*	0.04	0.42	1.53	0.03	0.20	0.48	0.03	0.37	1.46
Method *D*	0.43	1.14	-	0.25	0.50	-	0.35	1.06	-

**Elev. Angle**	**Maximal XYZ [m]**			**Maximal XY [m]**			**Maximal Z [m]**		
		
	**10°**	**30°**	**40°**	**10°**	**30°**	**40°**	**10°**	**30°**	**40°**

Method *A*	-	3.44	8.18	-	1.25	5.43	-	3.34	6.12
Method *B*	2.00	17.88	50.20	1.30	11.70	8.09	1.83	16.78	49.69
Method *C*	1.30	5.63	10.26	0.82	2.39	3.52	1.05	5.50	9.64
Method *D*	4.69	6.13	-	1.86	3.84	-	4.55	5.47	-
